# Hesperidin from Chenpi Ameliorates Skin Photoaging by Targeting HSPA1L to Stabilize GPX4 and Suppress Ferroptosis

**DOI:** 10.3390/antiox15040484

**Published:** 2026-04-14

**Authors:** Xiaoyu Guo, Mengyao Wu, Yunxing Li, Jianlang He, Yongjie Ma, Taizhi Su, Changzheng Li, Jian Wang

**Affiliations:** 1 Research Centre of Basic Integrative Medicine, School of Basic Medical Sciences, Guangzhou University of Chinese Medicine, Guangzhou 510006, China; 20201120123@stu.gzucm.edu.cn (X.G.); wumengyao@stu.gzucm.edu.cn (M.W.); liyunxingxing@163.com (Y.L.); 13690538781@163.com (J.H.); mayongjie@stu.gzucm.edu.cn (Y.M.); 13940157472@163.com (T.S.); 2 Department of Hematology, Guangzhou Medical University, Guangzhou 511436, China

**Keywords:** hesperidin, ferroptosis, HSPA1L, GPX4, LiP-MS, skin organoids

## Abstract

Photoaging is an extrinsic skin aging process caused by chronic ultraviolet (UV) radiation. A core pathological feature of photoaging is excessive oxidative stress, which can further induce ferroptosis. The HSP70 family plays a critical role in this stress response by protecting the key antioxidant enzyme GPX4. In this study, we established UV-induced photoaging models in cultured cells and 3D skin organoids. UPLC-MS/MS analysis of Chenpi transdermal permeate (prepared by in vitro transdermal penetration of Chenpi extract through mouse skin) identified hesperidin as the primary bioactive compound of Chenpi (dried peel of the plant *Citrus reticulata* Blanco after the aging process). The efficacy of hesperidin was validated in human keratinocytes (HaCaTs), fibroblasts (HSFs), and skin organoids. Mechanistically, transcriptomic and metabolomics analysis indicated that ferroptosis is a key pathway through which hesperidin ameliorates photoaging. Limited proteolysis mass spectrometry (LiP-MS), transcriptomics, and molecular dynamics simulation results demonstrated that hesperidin directly binds to the molecular chaperone HSPA1L. By upregulating HSPA1L expression, hesperidin enhanced the stability of GPX4 and suppressed UV-triggered ferroptosis. Our findings identify the HSPA1L/GPX4 axis as a critical redox regulatory pathway targeted by hesperidin, providing a mechanistic foundation for anti-photoaging therapies.

## 1. Introduction

Skin photoaging is a form of chronic cutaneous damage resulting from prolonged exposure to ultraviolet (UV) radiation. Its clinical features mainly include wrinkle formation, skin laxity, increased roughness, and severe loss of elasticity [1]. At the molecular level, UV radiation, particularly UVA and UVB, stimulates excessive intracellular reactive oxygen species (ROS) production [2]. This triggers sustained oxidative stress and inflammatory responses. UV irradiation also upregulates the expression and activity of matrix metalloproteinases (MMPs), including MMP-1, MMP-3 and MMP-9 in skin, which degrade and disrupt collagen fibers, ultimately causing structural and functional abnormalities in skin [3,4].

Recent studies have demonstrated that ferroptosis, a newly identified type of regulated cell death, contributes critically to the pathogenesis of photoaging [5]. Characterized by iron-dependent lipid peroxide accumulation, ferroptosis is closely associated with oxidative stress [6]. Accumulating evidence indicates that UVB radiation triggers ferroptosis in epidermal keratinocytes, which in turn accelerates cellular senescence and initiates inflammatory responses, thereby acting as a significant contributor to the photoaging phenotype [7]. Inhibition of ferroptosis effectively alleviates UVB-induced photoaging in keratinocytes [8]. Upon UVB exposure, ferroptosis is activated in HaCaT cells, which promotes inflammatory responses by inducing the release of high-mobility group box 1 (HMGB1) [9]. UV irradiation stimulates ROS production and induces mitophagy. The autophagy-related gene ATG5 promotes the degradation of glutathione peroxidase 4 (GPX4), thereby aggravating UVB-induced ferroptosis in keratinocytes. This cascade leads to interferon-gamma (IFN-γ) secretion and M1 macrophage polarization, ultimately worsening skin inflammation [10]. GPX4 is a key antioxidant enzyme that strongly suppresses lipid peroxidation. It functions as a central regulator of ferroptosis and participates in the modulation of cellular senescence, apoptosis, and cell death. Thus, GPX4 represents a critical target in antitumor therapy [11]. Therefore, strategies targeting ferroptosis inhibition or GPX4 activation show great potential for ameliorating skin aging. Studies have confirmed that suppressing ferroptosis and lipid peroxidation, while upregulating GPX4 expression, can delay both intrinsic aging and photoaging. These findings highlight the therapeutic value of targeting the ferroptosis pathway in the intervention of skin aging [12,13].

Heat shock protein 70 (HSP70) exerts critical protective effects against skin photoaging. HSP70 is a highly conserved protein induced by cellular stress. As a major molecular chaperone, HSP70 facilitates proper protein folding, assembly, and translocation. It also prevents stress-induced protein denaturation and aggregation. Previous studies have demonstrated that elevated HSP70 expression can reduce cellular oxidative stress and markedly improves the viability of mouse fibrosarcoma cells (WEHI-S) following UVB (290–320 nm) irradiation [14]. Consistent with this, recent research suggests that extracellular vesicles containing HSP70 may promote skin rejuvenation by increasing HSP70 expression, decreasing MMPs expression, and reducing oxidative stress in aged skin [15]. As a member of the HSP70 family, HSPA1L acts as a key molecular chaperone under stress conditions. It binds to substrate proteins and maintains their native conformation. This function prevents protein inactivation or degradation under proteotoxic stresses such as heat and oxidative stress [16,17]. Through this fundamental chaperone activity, HSPA1L contributes to the reduction in oxidative damage and lipid peroxidation, suggesting a potential indirect mechanism for suppressing ferroptosis. However, the specific role of HSPA1L in ferroptosis and its functional interaction with the key antioxidant enzyme GPX4 remain largely unknown.

Chenpi, the dried peel of Citrus reticulata Blanco, is a widely used traditional Chinese medicine with well-documented anti-inflammatory and antioxidant activities. Currently, natural compounds have attracted increasing attention in anti-photoaging research due to their multi-target effects and high safety. Among them, hesperidin is a flavonoid abundantly present in citrus fruits such as oranges, tangerines, and particularly enriched in Chenpi. It has demonstrated significant antioxidant, anti-inflammatory, and anti-apoptotic properties [18,19]. Our preliminary work has confirmed that Chenpi exhibits protective effects against skin photoaging [20]. However, whether hesperidin is the key active component underlying the anti-photoaging activity of Chenpi remains unclear. Furthermore, the detailed molecular mechanisms, especially the potential association with ferroptosis and the critical antioxidant enzyme GPX4, have not yet been explored.

In recent years, skin organoids have emerged as a powerful and physiologically relevant experimental model for skin research [21]. Skin organoids are self-organizing 3D cell aggregates derived from skin stem cells or primary skin cells. They can recapitulate the key structural and functional characteristics of native skin, including the intact epidermal–dermal junction, epidermal keratinocyte stratification, dermal fibroblasts, and extracellular matrix (ECM) components such as collagen and elastic fibers [22,23,24]. Unlike 2D cell cultures, skin organoids closely mimic the in vivo skin microenvironment. This enables the study of cell–cell and cell-ECM interactions, which are essential for maintaining skin homeostasis and mediating photoaging. Compared with animal models, skin organoids avoid species differences and ethical issues, and enable more precise and efficient evaluation of the efficacy and mechanism of bioactive agents in a human-relevant context. Given these advantages, skin organoids were employed in the present study to validate the anti-photoaging effects of hesperidin. This model allowed us to comprehensively assess the protective effects of hesperidin on both epidermal and dermal layers in a 3D physiological context, bridging the gap between 2D cell experiments and potential in vivo studies.

This study used a multi-level experimental strategy combining in vitro and skin organoid models to comprehensively investigate the anti-photoaging effects of Chenpi and its active component, hesperidin. Transcriptomics, lipidomics, limited proteolysis-mass spectrometry (LiP-MS), molecular docking, and molecular dynamics simulation were applied to explore the underlying molecular mechanisms. It aimed to clarify: (1) Hesperidin is the core active component in the transdermal permeate of Chenpi. (2) Hesperidin ameliorates UV-induced photoaging in epidermal cells, dermal fibroblasts, and skin organoids. (3) Hesperidin increases HSPA1L expression, maintains GPX4 protein stability, reduces UV-induced GPX4 degradation, and ultimately alleviates ferroptosis and photoaging. This study is the first to identify a novel association between hesperidin and the HSPA1L-mediated ferroptosis pathway. These findings highlight the potential of using natural products to develop novel interventions against skin photoaging.

## 2. Materials and Methods

### 2.1. Main Reagents and Instruments

Chenpi (purchased from Dashenlin Pharmaceutical Group Co., Ltd., Guangzhou, China), Hesperidin (HPLC ≥ 98%, A0032, Must Biotechnology Co., Ltd., Chengdu, China), RSL3 (HPLC ≥ 99.25%, HY-100218A, MedChemExpress, Shanghai, China), Ferrostatin-1 (HPLC ≥ 99.71%, HY-100579, MedChemExpress, Shanghai, China), DEL-I25 (HY-100579, MedChemExpress, Shanghai, China), Cycloheximide (HPLC ≥ 99.82%, HY-12320, MedChemExpress, Shanghai, China), DMEM/F12 (10-092-CVRC, Corning, Nanjing, China), Fetal bovine serum (164210, Procell, Wuhan, China), trypsin-EDTA (C100C1, NCM Biotech, Suzhou, China), RNA purification kit (B0004D, EZBioscience, Roseville, CA, USA), Color reverse transcription kit (A0010CGQ, EZBioscience, Roseville, CA, USA), 2× SYBR Green qPCR Master Mix (A0001-R2, EZBioscience, Roseville, CA, USA), RIPA lysis buffer (Beyotime Biotechnology, Shanghai, China), PMSF (36978, Thermo Fisher Scientific, Waltham, MA, USA), BCA protein assay kit (P0009, Beyotime, Shanghai, China), Sodium Dodecyl Sulfate-polyacrylamide Gel Electrophoresis (PG112, Epizyme Biotech, Shagnhai, China), PVDF membranes (ISEQ00010, Millipore, Burlington, NC, USA), anti- COL1A1 antibody (sc-293182, Santa Cruz Biotechnology, CA, USA), anti-Collagen III antibody (ab184993, Abcam, Cambridge, UK), anti- Glutathione Peroxidase 4 (61599, Genuinbiotech, Hefei, China), anti-Solute Carrier Family 7 Member 11 (2775, Genuinbiotech, Hefei, China), anti-GAPDH (66004-1-Ig, Proteintech, Wuhan, China), anti-β-actin (66009-1-Ig, Proteintech, Wuhan, China), Nrf2 Antibody (AF0639, Affinity, Liyang, China), Tris-EDTA buffer (Servicebio, Cat#G1206-250ML, pH = 8.0, Wuhan, China), anti-CD68 Rabbit pAb (Servicebio, GB113109-100), anti-K14 monoclonal antibody (AF-5370, Affinity, Liyang, China), anti-COL3 polyclonal antibody (Abcam, ab184993), and anti-SA-β-gal polyclonal antibody (Proteintech, Cat No. 15518-1-AP, Wuhan, China), TruePrep™ DNA Library Preparation Kit V2 (TD503, Vazyme, Nanjing, China), Qubit dsDNA HS Assay Kit (Q32854, Invitrogen, Carlsbad, CA, USA), Reactive Oxygen Species Detection Kit (Solarbio, CA1410, Beijing, China), Dihydroethidium (S0064S, Beyotime, Shanghai, China), β-galactosidase staining solution (C0602, Beyotime, Shanghai, China), IL-1β, IL-6, and TNF-α ELISA kit (E-HSEL-H0001/E-HSEL-H0003/E-EL-H0109, ElabScience, Wuhan, China), CCK-8 assay kit (C6005, NCM Biotech, Suzhou, China), Mitochondrial Superoxide Detection Kit (S0061S, Beyotime, Shanghai, China), GSH and GSSG Assay Kit (S0053, Beyotime, Shanghai, China), VAHTS RNA Clean Beads (N412, Vazyme, Nanjing, China), VAHTS DNA Clean Beads (N411, Vazyme), TruePrep™ DNA Library Preparation Kit V2 (TD503, Vazyme), protease and phosphatase inhibitors (P0013, P1045 Beyotime, Shanghai, China), GENEWIZ (GENEWIZ, Suzhou, China), DNA Oligos (D0251, Beyotime, Shanghai, China), pLKO.1-EGFP vector (P0255, MiaoLing, Wuhan, China), HiPure Plasmid EF Mini Kit (P211, Magen, Guagnzhou, China), Lipo8000™ Transfection Reagent (C0533-0.5mL, Beyotime, Shanghai, China), Ferric and Ferrous Ion Assay Kit (Beyotime, S1066, Shanghai, China), MDA Assay Kit (KTB1050, Abbkine, Wuhan, China), SOD activity assay kit (Abbkine, Cat# KTB1030), Nuclear and Cytoplasmic Protein Extraction Kit (P0027, Beyotime), UVA and UVB lamps (YIXIAN Medical, Dongguan, China), Chemiluminescence instrument (Tanon, Shanghai, China), Laser Confocal Microscope (ZEISS, Oberkochen, German), Inverted fluorescence microscope (Bio-rad, ZOE, Hercules, CA, USA), inverted microscope (Olympus, Tokyo, Japan), multimode microplate reader (Synergy Neo2, Agilent, Waltham, CA, USA), FEI Tecnai SPIRIT transmission electron microscope (FEI, Eindhoven, The Netherlands), GATAN CCD camera (GATAN, 832.10), Orbitrap Exploris™ 480 mass spectrometer (Thermo Fisher Scientific, USA), QExactive mass spectrometer (Thermo Fisher Scientific, USA), Thermo Scientific EASY-nLC™ 1200 system (Thermo Fisher Scientific, USA), Orbitrap Exploris™ 480 mass spectrometer (Thermo Fisher Scientific, USA), FEI Tecnai SPIRIT transmission electron microscope (Thermo Fisher Scientific, USA).

### 2.2. Cell Culture

HSF cells were purchased from Fuheng Biology (FH0189, Shanghai, China) and cultured in DMEM/F12 medium supplemented with 15% fetal bovine serum 100 U/mL penicillin, and 100 μg/mL streptomycin. HaCaTs were purchased from Wuhan Procell Biotechnology (CL-0090, Wuhan, China) and cultured in high-glucose DMEM medium containing 15% fetal bovine serum, 100 U/mL penicillin, and 100 μg/mL streptomycin. Cells were cultured in a humidified incubator at 37 °C and 5% CO_2_, and passaged using trypsin-EDTA. Experiments were conducted using cells from the 3rd to 10th passages.

### 2.3. Animals

BALB/c-nu female mice, aged 8 weeks, were procured from Zhuhai Bestone Biotechnology Co., Ltd. (Zhuhai, China) (Licence No. SYXK [Yue] 2020-0051). The animals were maintained under standard housing conditions and received no experimental treatment. Animal use and experimental procedures were approved by the Animal Care and Use Committee of Guangzhou University of Chinese Medicine (Approval number: No. 20251126011).

### 2.4. Establishment of Cellular Photoaging Model and Drug Administration

HaCaT and HSF cells were seeded in 96-well plates at a density of 1 × 10^5^ cells/mL. After 24 h of incubation for cell attachment, the cells were exposed to UV irradiation for 3 consecutive days. The total irradiation doses were 120 mJ/cm^2^ UVA and 80 mJ/cm^2^ UVB. Cells in the treatment groups were incubated with their respective medium containing: high-dose hesperidin (10 μM), low-dose hesperidin (5 μM), vitamin E (0.5% *v*/*v*), naringin (10 μM), nobiletin (10 μM), sinensetin (10 μM), or Chenpi transdermal permeate fluid (10% *v*/*v*). After 24 h of incubation, samples were collected for subsequent analysis.

### 2.5. Organoid Construction and Modeling with Drug Administration

The skin organoid culture method was performed as previously described [23,25], with minor modifications. Skin tissues were isolated from 24 h-old C57BL/6 mice. The epidermis and dermis were separated following enzymatic digestion. Each layer was minced and digested to obtain single-cell suspensions. Then the epidermal and dermal cells were mixed and seeded onto transwell inserts for 3D organoid culture. On day 7, organoids were exposed to UV irradiation for 3 consecutive days at a daily dose of 80 mJ/cm^2^ UVB and 160 mJ/cm^2^ UVA. After treatment, samples were collected and fixed in 4% paraformaldehyde (PFA) for 24 h. Fixed samples were then sectioned and subjected to immunostaining or other marker analyses. Culture supernatants were collected for ELISA assays.

### 2.6. Reverse Transcription Quantitative Polymerase Chain Reaction (RT-qPCR)

Total RNA of HaCaT and HSF cells was extracted from samples using a commercial RNA purification kit. RNA concentration and purity were measured prior to reverse transcription. A total of 1000 ng of qualified RNA was reverse-transcribed into cDNA using a color reverse transcription kit. Real-time quantitative PCR (qPCR) was performed using 2 × SYBR Green qPCR Master Mix. Relative mRNA expression was calculated using the 2^−ΔΔCt^ method and normalized to GAPDH. Fold changes were determined relative to the control group. Primer sequences are listed in Supporting Information (Table S1).

### 2.7. Western Blotting (WB)

Total protein of HaCaT, HSF cells and skin tissue was extracted using RIPA lysis buffer supplemented with PMSF at a ratio of 100:1. Nucleoproteins were isolated using a commercial kit. Protein concentration was determined using a BCA protein assay kit. β-actin or GAPDH was used as the internal control. H3 was used as the internal control for the nucleoprotein. Protein samples were separated by sodium dodecyl sulfate-polyacrylamide gel electrophoresis (SDS-PAGE) and transferred onto polyvinylidene fluoride (PVDF) membranes. Incubate the membrane with the following primary antibody overnight at 4 °C: anti-COL1A1 antibody, anti-Collagen III antibody, anti-Glutathione Peroxidase 4, anti-Solute Carrier Family 7 Member 11, anti-Nrf2, anti-GAPDH, anti-β-actin, anti-H3. Secondary antibody was pre-labelled at room temperature for 1 h. Protein bands were visualized using enhanced chemiluminescence (ECL) reagent. Results were quantified using ImageJ software (Version 1.53t).

### 2.8. Immunofluorescence

Skin and organoid samples were fixed in 4% PFA for 24 h and then embedded in paraffin. Paraffin sections (4 μm) were deparaffinized and heated in Tris-EDTA buffer for antigen retrieval. After washing, sections were blocked in PBS containing 5% BSA for 1 h at room temperature. Sections were then incubated overnight at 4 °C in the dark with the following primary antibodies: anti-CD68 rabbit pAb, anti-K14 monoclonal antibody, anti-COL3 polyclonal antibody, and anti-SA-β-gal polyclonal antibody. After washing, sections were incubated with fluorescence-conjugated secondary antibodies for 1 h at room temperature in the dark. Finally, the sections were stained with DAPI for 10 min at room temperature and mounted with fluorescent mounting medium. Images were captured using a laser confocal microscope.

### 2.9. ROS Staining and Quantification

Cellular staining: Intracellular ROS production in HaCaT and HSF cells was measured using the Reactive Oxygen Species Detection Kit. Cells were incubated with DCFH-DA at 37 °C for 30 min, washed three times, stained with Hoechst for nuclear labeling. Fluorescent images were captured using an inverted fluorescence microscope. Fluorescence intensity was quantified using ImageJ software.

Organoids: Intracellular ROS levels in skin organoids were measured using dihydroethidium (DHE). Paraffin sections were incubated with DHE at 37 °C for 20 min. Sections were then mounted with DAPI-containing mounting medium. Images were acquired using a laser confocal microscope. Fluorescence intensity was quantified using ImageJ.

### 2.10. SA-β-Gal Staining

HSF cells were washed three times with PBS and fixed for 15 min at room temperature. After fixation, cells were washed another three times with PBS. Cells were then incubated overnight at 37 °C in freshly prepared β-galactosidase staining solution, protected from light. Images were captured using an inverted microscope. The percentage of positive staining cells was quantified and analyzed using ImageJ software.

### 2.11. Cell Migration

The migratory capacity of HaCaT cells was evaluated using scratch wound healing assays. HaCaT cells were seeded into 6-well plates at a density of 1 × 10^5^ cells per well. After 24 h of attachment, a linear scratch was created in the cell monolayer using a 200 μL pipette tip. Cells were washed with PBS to remove detached cell debris. Images were captured at 0, 24, and 48 h using an inverted microscope. The remaining wound width was measured using ImageJ software. The migration rate was calculated as the ratio of the migrated area to the initial scratch area.

### 2.12. ELISA

Culture supernatants were collected from HSF cells, HaCaT cells, and skin organoids. Supernatants were centrifuged at 3000 rpm for 10 min at 4 °C. The levels of IL-1β, IL-6, and TNF-α were measured according to the manufacturer’s instructions.

### 2.13. Cell Counting Kit-8 (CCK-8)

Cell viability of HaCaT and HSF cells was assessed using a CCK-8 assay kit. Cells were seeded into 96-well plates at a density of 5 × 10^4^ cells per well. After UV irradiation, cells were incubated for 24 h. CCK-8 reagent was added to serum-free medium at 100 μL per well. After incubation at 37 °C for 0.5–1 h, absorbance at 450 nm was detected using a multimode microplate reader.

### 2.14. mtROS Assay

Mitochondrial ROS production in HaCaT cells was detected using a Mitochondrial Superoxide Detection Kit. Cells were incubated with DCFH-DA at 37 °C for 30 min. After three washes, cells were stained with Hoechst for nuclear labeling. Fluorescent images were captured using an inverted fluorescence microscope. Fluorescence intensity was quantified using ImageJ software.

### 2.15. GSH and GSSG Assay

The ratio of glutathione (GSH) to glutathione disulfide (GSSG) of HaCaT cells was determined using a commercial GSH and GSSG assay kit. HaCaT cells were lysed according to the kit instructions. After high-speed centrifugation, the supernatant was collected for GSH and GSSG detection. The GSH/GSSG ratio was then calculated.

### 2.16. Detection of Intracellular Fe^2+^ Concentration

Intracellular Fe^2+^ concentrations of HaCaT cells were measured using a commercial Ferric and Ferrous ion assay Kit. Briefly, HaCaT cells were lysed in the provided BeyoLysis™ buffer and centrifuged to collect the supernatant. Assays were performed according to the manufacturer’s protocol. Fe^2+^ concentrations were calculated from a standard curve and normalized to the total protein content of each sample.

### 2.17. Measurement of Malondialdehyde (MDA) Content

The lipid peroxidation product MDA was quantified using a commercial MDA assay kit following the manufacturer’s protocol. Briefly, HaCaT and HSF cells were homogenized in ice-cold Extraction Buffer and centrifuged at 13,000× *g* for 10 min at 4 °C to obtain the supernatant. For the assay, 100 µL of the sample supernatant was mixed with 300 µL of Reaction Mix. The mixture was incubated at 95 °C for 30 min, cooled on ice, and centrifuged at 10,000× *g* for 10 min. The absorbance of the supernatant was measured at 532 nm and 600 nm using a microplate reader. The MDA concentration was calculated based on the absorbance difference (ΔA = A_532_ − A_600_) and normalized to the total protein concentration of the sample, determined by a BCA assay.

### 2.18. Measurement of Superoxide Dismutase (SOD) Activity

SOD activity was measured using a commercial SOD activity assay kit following the manufacturer’s instructions. Briefly, HaCaT and HSF cells were homogenized in ice-cold lysis buffer, followed by centrifugation. The resulting supernatant was collected for the assay. In a 96-well plate, 20 µL of sample was mixed with 164 µL of Working Reagent and 40 µL of diluted xanthine oxidase. After incubation at 37 °C for 30 min, the absorbance was measured at 450 nm using a microplate reader. SOD activity was determined by calculating the percentage inhibition of the WST-8 formazan formation. The final activity was normalized to the total protein concentration of the sample, which was determined in parallel using a BCA protein assay kit.

### 2.19. Preparation of Transdermal Fluid

Chenpi (10.0 g) was accurately weighed and extracted with 20 volumes of 70% ethanol under sonication for 30 min. The mixture was filtered, and the residue was further extracted with an equal volume of 50% ethanol under sonication for 30 min and filtered again. The residue was then extracted with an equal volume of 30% ethanol under sonication for 30 min and filtered. The residue was discarded, and all filtrates were combined and concentrated under reduced pressure until no ethanol odor was detected. PEG 4000 (2.5 mL) was added, and the mixture was shaken for 2 min to dissolve insoluble substances. The solution was diluted to 250 mL with distilled water in a volumetric flask.

Abdominal skin samples were obtained from BALB/c-nu mice that had not undergone any experimental treatment. Subcutaneous fascia and connective tissues were removed with surgical scissors. The skin was rinsed repeatedly with physiological saline. The prepared skin was cut to an appropriate size and fixed onto a YB-P6 transdermal diffusion cell. The diffusion cell had a volume of 10 mL and an effective permeation area of 1.13 cm^2^. The skin was mounted with the epidermis facing upward and the dermis facing downward. A 40% ethanol solution was used as the receiving medium. The prepared Chenpi solution (5 mL) was added to the donor chamber. The diffusion system was maintained in an intelligent transdermal apparatus at 37 °C and 150 r/min for 24 h. The receiving solution was collected and evaporated to dryness in a 50 °C water bath. The residue was reconstituted with 5 mL of culture medium to obtain the Chenpi transdermal permeation solution. Osmotic pressure was measured using a freezing-point osmometer (Advanced Instruments, Norwood, MA, USA) and was consistent with physiological saline. The solution was sterilized by filtration through a 0.22 μm filter prior to UPLC-MS/MS analysis.

### 2.20. RNA Sequencing and Data Analysis

HaCaT cells were seeded in 6-well plates at a density of 1 × 10^5^ cells per well. After 24 h of attachment, cells were exposed to UV irradiation and treated with the corresponding reagents. Following 24 h of incubation, the medium was removed, and cells were washed with PBS. Total RNA was extracted by adding 1 mL of Trizol reagent to each well.

RNA was purified using 80 μL VAHTS RNA Clean Beads. Reverse transcription was performed with 5 μL purified RNA and 15 μL RT mix. cDNA was purified via VAHTS DNA Clean Beads and subjected to 12 cycles of PCR amplification. The purified cDNA was quantified using the Qubit dsDNA HS Assay Kit. RNA-sequencing libraries were prepared using the TruePrep™ DNA Library Preparation Kit V2 according to the manufacturer’s instructions. Libraries were sequenced on the NovaSeq X Plus PE150 platform. Each group included at least three biological replicates.

### 2.21. Metabolomic

Lipid metabolites in HaCaT cells were analyzed. Lipids were extracted using a classical water/chloroform/methanol method. Briefly, methanol, water, and chloroform were sequentially added to cell pellets at a final volume ratio of 5:2:5. The chloroform phase was collected and dried under nitrogen gas at room temperature. Dried lipids were re-dissolved in a chloroform/methanol/water mixture (6:3:0.5, *v*/*v*). Liquid chromatography was performed using an Ultimate 3000 LC system coupled with a QExactive mass spectrometer. Metabolomics liquid chromatography-mass spectrometry analysis was conducted on a Hyperil Gold C18 column at 40 °C with a flow rate of 0.30 mL min^−1^. Mass spectrometry was performed on the QExactive system. Data-dependent tandem mass spectrometry (MS/MS) was performed with secondary resolution of 17,500, top ten, collision energy of 20 and 40, and a mass range of 70–1500 m.z. Raw lipidomics data were processed using LipidSearch 5.1 software for lipid identification and comparative analysis. Lipid annotation was performed in daughter ion mode using full-spectrum and data-dependent MS/MS data acquired by the QExactive system.

### 2.22. Molecular Docking and Molecular Dynamics Simulations

The 3D SDF (Structure Data File) for Hesperidin downloaded from PubChem (Compound CID: 10621). The three-dimensional crystal structure of the HSPA1L complex was obtained from the Protein Data Bank (PDB) (http://www.rcsb.org, PDB ID: 3GDQ, accessed on 20 October 2025). Using Pymol, redundant structures, ligands, and surrounding water molecules were removed. Subsequently, molecular docking between hesperidin and HSPA1L was performed using CB-Dock2 (https://cadd.labshare.cn/cb-dock2/php/index.php, accessed on 21 October 2025). The most suitable docking conformation for subsequent molecular dynamics simulations was selected based on docking scores and alignment with the HSPA1L active site. Briefly, protein topology files were constructed using the AMBER99SB-ILDN force field and SPC/E water model, while ligand topology and parameter files were generated via the Acpype Server online platform (Acpype Server (bio2byte.be)). Subsequent steps included energy minimization, NVT and NPT equilibration, and 100 ns molecular dynamics simulations. For analysis of simulation results, we employed Gromacs 2020.6 software to assess RMSD, RMSF, particle radius of gyration (Rg), and Gibbs Free Energy Landscape. Visualization was performed using Pymol 2.6.0 software, with 3D Gibbs Free Energy Landscape visualized via DuIvyTools v0.6.0. Additionally, the MM/GBSA method in gmx_MMPBSA v1.6.2 (https://valdes-tresanco-ms.github.io/gmx_MMPBSA/dev/) was employed to calculate the binding free energy between hesperidin and HSPA1L, investigating ligand-enzyme affinity. Visualization was performed using PyMOL 2.6.0.

### 2.23. Limited Proteolysis Mass Spectrometry (LiP-MS)

Sample Preparation: HaCaT cells were seeded in 6-well plates at a density of 1 × 10^5^ cells per well. After 24 h of attachment, cells were lysed using a cell lysis buffer containing protease and phosphatase inhibitors. An appropriate sample volume was centrifuged at 20,000× *g* for 20 min at 4 °C. The supernatant was collected and protein quantification was performed using the BCA method. A portion of the sample was subjected to SDS-PAGE electrophoresis. Protein samples were divided into two groups and incubated with DMSO or hesperidin at 25 °C for 5 min. DTT was added to a final concentration of 10 mM, followed by incubation at 37 °C for 1 h. IAA was then added to a final concentration of 20 mM, and samples were incubated in the dark for 30 min. A 100 μL salt buffer solution was added to FASP tubes, followed by centrifugation at 13,900× *g* for 20 min at 15 °C. A total of 100 μg of reduced and alkylated protein was transferred to FASP tubes and centrifuged at 13,900× *g* for 20 min at 15 °C until all liquid passed through the filter membrane. Then, 200 μL of buffer was added, and tubes were centrifuged at 13,900× *g* for 20 min at 15 °C. This washing step was repeated three times. Trypsin was added at a protein-to-enzyme ratio of 75:1. FASP tubes were capped and incubated at 37 °C for 14–16 h. Tubes were transferred to collection tubes and centrifuged at 13,900× *g* for 20 min at 15 °C. A 100 μL solution of 75% acetonitrile with 0.1% formic acid was added, followed by centrifugation at 13,900× *g* for 20 min at 15 °C. This step was repeated once. Peptide samples were evaporated to dryness under vacuum and stored at −20 °C until analysis.

Mass Spectrometry Analysis (LC-MS/MS): Dried peptide samples were resuspended in 0.1% formic acid (FA) solution. After centrifugation at 20,000× *g* for 10 min, the supernatant was injected for analysis. Peptide separation was performed using a Thermo Scientific EASY-nLC™ 1200 system. Samples were loaded onto a self-packed C18 column. Peptides were eluted at a flow rate of 300 nL/min with the following gradient: 0–66 min: 5% to 27% mobile phase B (98% ACN, 0.1% FA); 66–75 min: 27% to 40% mobile phase B; 75–76 min: 40% to 90% mobile phase B; 76–90 min: 90% mobile phase B. Eluted peptides were ionized by nanoESI and analyzed using an Orbitrap Exploris™ 480 mass spectrometer in data-dependent acquisition (DDA) mode.

### 2.24. Transmission Electron Microscopy

Mitochondrial morphology in HaCaT cells was observed using transmission electron microscopy. Cells were washed with PBS and fixed in 2.5% glutaraldehyde at room temperature. Samples were post-fixed with osmium tetroxide and uranyl acetate. After dehydration using a graded acetone series, samples were embedded in epoxy resin. Ultrathin sections were prepared and stained with 2% uranyl acetate and 1% lead citrate. Sections were examined at 80 kV using a FEI Tecnai SPIRIT transmission electron microscope. Images were acquired using a GATAN CCD camera.

### 2.25. Microscale Thermophoresis (MST)

HaCaT cells were collected 48 h after transfection with the eGFP-labelled HSPA1L plasmid and lysed using a lysis buffer supplemented with protease and phosphatase inhibitors. HSPA1L expression was validated by total fluorescence intensity. For binding assays, lysates were diluted 4-fold in ECF buffer containing 0.1% Tween-20 to achieve optimal fluorescent protein levels. Compounds were titrated in ECF at a 1:1 ratio, diluted 16-fold. Subsequently, 5 μL of cell lysate was mixed with 5 μL of compound at varying concentrations. After incubation at room temperature for 5 min, all samples were loaded into MST NT.115 standard glass capillaries. Measurements were performed using MO Control software (v.1.6.1) with excitation power of 40–100% at 25 °C. Data were then imported into NanoTemper’s MO Affinity Analysis software (v.3.0.5) to calculate Kd values using the Kd model. Cold fluorescence and hot fluorescence were measured at −1 to 0 s and 19 to 20 s respectively to avoid heat-induced protein conformational changes. Curve fitting was performed using GraphPad Prism 9.2.0 software, with all Kd values calculated at a 95% confidence level.

### 2.26. Plasmid Construction and Transfection

Four complementary oligonucleotides encoding shRNA targeting human GPX4 and HSPA1L mRNA, with Age1 and EcoR1 overhangs, were designed to knock down the expression. Single-stranded oligonucleotides were synthesized by GENEWIZ. Forward and reverse oligonucleotides were annealed to form double-stranded oligomers using the Annealing Buffer for DNA Oligos according to the manufacturer’s protocol. The annealed DNA was ligated into the linearized pLKO.1-EGFP vector at the Age1 and EcoR1 sites. The constructed plasmids were verified by direct DNA sequencing. Plasmid DNA was isolated using the HiPure Plasmid EF Mini Kit. The sequences of sh-GPX4, sh-HSPA1L, OE-HSPA1L were listed in Supporting Information (Table S2).

After 24 h, transfection was performed using Lipo8000™ Transfection Reagent following the manufacturer’s instructions in HaCaT cells. Knockdown efficiency was validated by RT-qPCR.

### 2.27. Data Statistics and Analysis

Statistical analysis was performed using GraphPad Prism 9.2.0 software. Comparisons between groups were analyzed by one-way analysis of variance (ANOVA) followed by Tukey’s multiple comparisons test or Dunnett’s multiple comparisons test. For data that did not follow a normal distribution, nonparametric tests were used to analyze intergroup differences. Differences were defined statistically significant when * *p* < 0.05, ** *p* < 0.01, *** *p* < 0.001 and **** *p* < 0.0001. ps. *p* < 0.05 was considered statistically significant.

## 3. Results

### 3.1. Identification of Hesperidin as the Primary Penetrating Bioactive Component of Chenpi

**Figure 1:**
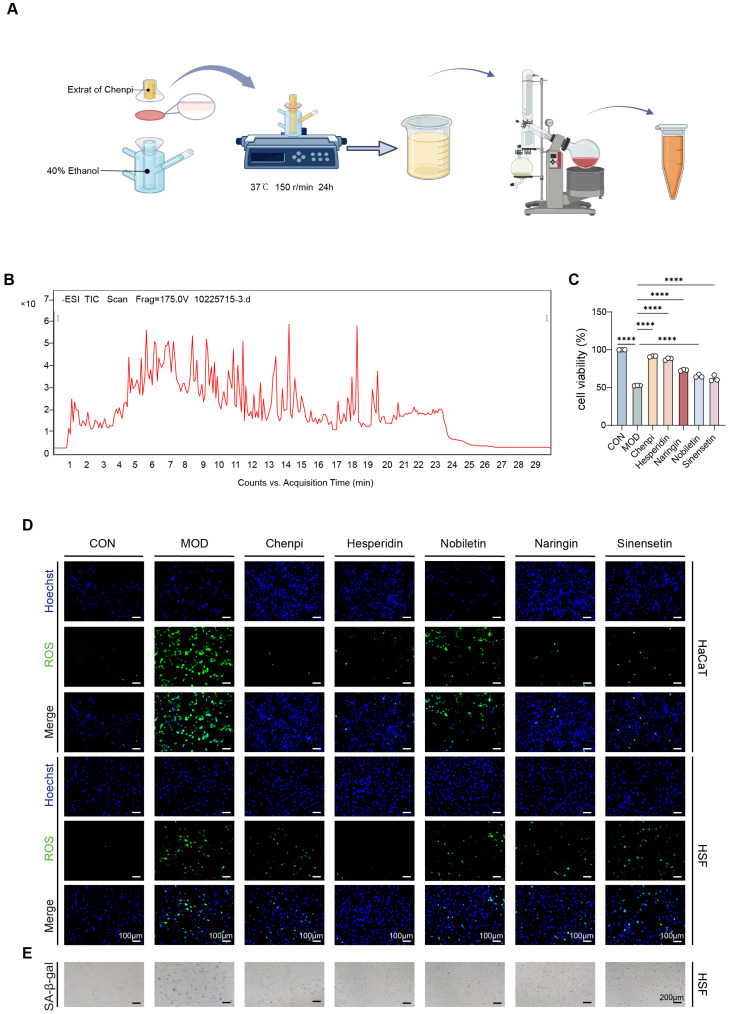
Hesperidin was the primary bioactive component in transdermal permeate of Chenpi. (**A**) Flowchart of Chenpi transdermal solution preparation. (**B**) Anion chromatography profile of Chenpi transdermal solution. (**C**) Cell viability after UV exposure and treatment of HaCaT cells. (scale bar = 100 μm) (**D**) ROS fluorescence staining of HaCaT cells and HSF cells. (ROS, green; Hoechst, blue) (scale bar = 100 μm) (**E**) SA-β-gal staining of HSF cells (scale bar = 200 μm). (*n* = 3, **** *p* < 0.0001).

Topical administration is a common strategy for the treatment of skin disorders. Compared with oral administration, skin penetration is critical for the therapeutic efficacy of topical agents and represents a prerequisite for their bioactivity. In previous studies, we observed that topical application of Chenpi significantly alleviated photoaging in a nude mouse model [20]. Therefore, to identify these penetrating anti-photoaging constituents from Chenpi, we performed an in vitro transdermal permeation assay. Compounds that permeated the skin were collected as the transdermal permeate and analyzed by ultra-performance liquid chromatography–tandem mass spectrometry (UPLC-MS/MS) (Figure 1A). UPLC-MS/MS profiling identified hesperidin as the most abundant component in the transdermal permeate (Figure 1B; Table 1). This finding identified it as the primary candidate responsible for the biological activity of the topically applied Chenpi. The skin is a complex multi-layered organ, and UV-induced photoaging affects both the epidermis and dermis. Accordingly, we selected two cell lines representing the epidermis and dermis to evaluate the protective effects of Chenpi transdermal fluid and its major components against UV-induced photoaging. Using the model of UV-induced damage in human keratinocytes (HaCaT) and skin fibroblasts (HSF), we found that both Chenpi transdermal permeate and the active ingredients significantly improved cell viability after irradiation (Figure 1C). Notably, hesperidin exhibited the most potent effect, with efficacy comparable to that of the total transdermal fluid. UV irradiation triggers oxidative stress in the skin, accompanied by excessive ROS accumulation. Subsequent experiments confirmed that hesperidin effectively reduced UV-induced ROS production in both HaCaT and HSF cells (Figure 1D). Furthermore, hesperidin significantly decreased the proportion of senescence-associated β-galactosidase (SA-β-gal)-positive HSF cells, a hallmark of stress-induced cellular senescence (Figure 1E). Collectively, these results demonstrate that hesperidin is the major bioactive component of Chenpi transdermal fluid. It recapitulates the cytoprotective, antioxidant, and anti-senescent activities of the crude transdermal permeate against UV-induced photoaging in skin cells. To ensure mechanistic clarity, specificity, and reproducibility, purified hesperidin (rather than crude transdermal permeate) was used for all subsequent in-depth mechanistic experiments. This approach allows unambiguous attribution of the observed molecular effects to hesperidin, the key skin-available active principle of topically administered Chenpi.

### 3.2. Hesperidin Alleviated UV-Induced Photoaging Damage in HaCaT Cells

**Figure 2:**
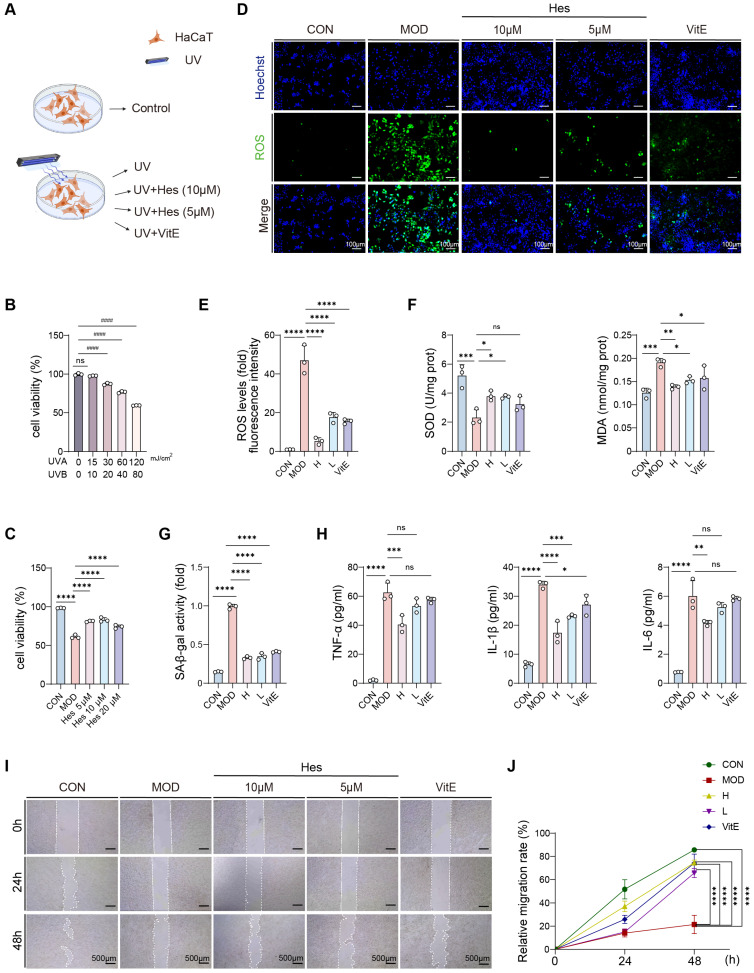
Hesperidin ameliorated photoaging in HaCaT cells. (**A**) Cell treatment and administration workflow. (**B**) Cell viability after UV exposure. (**C**) Cell viability after UV exposure and hesperidin treatment. (**D**) ROS fluorescence staining in HaCaT cells (ROS, green; Hoechst, blue) (scale bar = 100 μm). (**E**) Quantitative analysis of ROS fluorescence staining (fold with control). (**F**) Cellular MDA and SOD levels. (**G**) SA-β-gal activity (fold with control). (**H**) IL-1β, IL-6, TNF-α concentration in cell supernatants. (**I**) Representative images of migration assay (scale bar = 500 μm). (**J**) Statistical analysis of 24–48 h cell migration rate. (*n* = 3, compared with the model group: * *p* < 0.05, ** *p* < 0.01, *** *p* < 0.001, **** *p* < 0.0001; compared with the control group: #### *p* < 0.0001; ns: not statistically significant).

**Figure 3:**
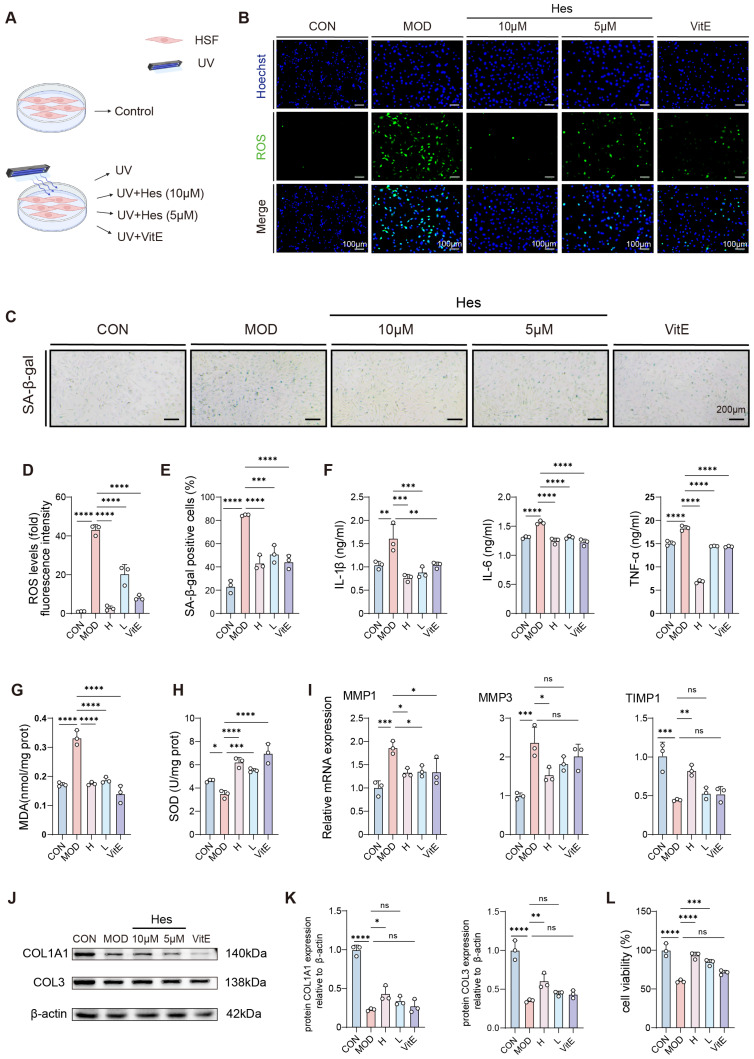
Hesperidin relieved photoaging in HSF cells. (**A**) Cell treatment and administration workflow. (**B**) ROS fluorescence staining in HSF cells (ROS, green; Hoechst, blue) (scale bar = 100 μm). (**C**) SA-β-gal staining (scale bar = 200 μm). (**D**) Quantitative analysis of ROS fluorescence staining (fold with control). (**E**) Statistical analysis of SA-β-gal staining positivity rate. (**F**) IL-1β, IL-6, TNF-α concentration in cell supernatants. (**G**) Cellular MDA level. (**H**) Cellular SOD activity level. (**I**) Quantitation of mRNA levels of MMP-1, MMP-3, and TIMP1. (**J**,**K**) Western Blot analysis of COL1A1 and COL3 protein expression levels. (**L**) Cell viability after UV exposure and hesperidin treatment. (*n* = 3, * *p* < 0.05, ** *p* < 0.01, *** *p* < 0.001, **** *p* < 0.0001; ns: not statistically significant).

Given that hesperidin was identified as the main bioactive component in the transdermal permeate, we further evaluated its cytoprotective effects against UV-induced photoaging (Figure 2A and Figure 3A). To establish a stable photoaging cell model, we first determined the optimal UV irradiation dose by measuring HaCaT cell viability using the CCK-8 assay. Cell viability gradually decreased with increasing UV dosage. At a UVA/UVB dose of 120/80 mJ/cm^2^, cell viability was significantly reduced to approximately 50% (*p* < 0.0001) (Figure 2B), indicating this dose was suitable for subsequent intervention studies. Next, we verified the cytoprotective effects of hesperidin. CCK-8 assays showed that hesperidin significantly increased the viability of photoaged HaCaT cells, with the strongest effect observed at 10 μM (Figure 2C). We then explored the underlying antioxidant mechanism. Hesperidin treatment inhibited the production of UV-induced intracellular ROS in a dose-dependent manner (Figure 2D,E). Meanwhile, hesperidin reduced the accumulation of malondialdehyde (MDA), a lipid peroxidation product, and increased the activity of the antioxidant enzyme superoxide dismutase (SOD) in photoaging cells (Figure 2F). These results indicate that hesperidin attenuates oxidative stress and lipid damage while enhancing endogenous antioxidant capacity. Furthermore, hesperidin significantly decreased the elevated SA-β-gal activity induced by UV irradiation, confirming its ability in alleviating UV-induced cellular senescence (Figure 2G). UV radiation triggers skin inflammation, and both oxidative stress and cellular senescence promote inflammatory cytokine release. Consequently, we further investigated the anti-inflammatory effects of hesperidin. ELISA results demonstrated that hesperidin markedly reduced the levels of TNF-α, IL-1β, and IL-6 in the supernatants of photoaged HaCaT cells (Figure 2H), suggesting an anti-inflammatory role. Finally, scratch-wound healing assays were performed to evaluate epidermal repair function. Hesperidin significantly improved the migration rate of HaCaT cells compared with the model group (Figure 2I,J), indicating enhanced epidermal barrier repair after photodamage.

In conclusion, these results demonstrate that hesperidin exerts comprehensive protective effects on UV-irradiated keratinocytes by improving cell viability, inhibiting oxidative stress and lipid peroxidation, reducing cellular senescence and inflammation, and promoting migratory repair.

### 3.3. Hesperidin Ameliorated Photoaging of HSF Cells

We further examined whether hesperidin protects human dermal fibroblasts (HSF), which are critical for dermal structure and extracellular matrix (ECM) synthesis. Consistent with its effect in HaCaT cells, hesperidin dose-dependently reduced UV-induced intracellular ROS production in HSF cells (Figure 3B,D). This reduction in oxidative stress was accompanied by a decreased proportion of SA-β-gal-positive senescent cells (Figure 3C,E), supporting that hesperidin alleviates UV-induced premature senescence. The alleviation of cellular senescence was functionally linked to a reduced inflammatory response. Compared with the model group, hesperidin significantly reduced the secretion of IL-6, TNF-α, and IL-1β (Figure 3F), indicating suppression of the senescence-associated secretory phenotype (SASP). Consistent with the effects observed in epidermal cells (Figure 2F), both high and low doses of hesperidin actively enhanced the cellular antioxidant defense system by significantly elevating the activity of SOD, and reduced MDA production (Figure 3G,H). This enhanced antioxidant capacity favored balanced remodeling of the dermal ECM. Subsequently, we investigated how hesperidin coordinates the dermal repair process. Hesperidin downregulated mRNA expression of the matrix metalloproteinases MMP-1 and MMP-3, while upregulating their inhibitor TIMP-1 (Figure 3I), thus reducing ECM degradation. Western blot analysis confirmed that hesperidin increased protein levels of type I and type III collagens (COL1A1 and COL3A1) (Figure 3J,K), which are essential for skin elasticity and structural integrity. Furthermore, hesperidin dose-dependently improved the viability of UV-irradiated HSF cells (Figure 3L). Generally speaking, these results indicate that hesperidin attenuates UV-induced damage by suppressing oxidative stress, inflammation, and cellular senescence, while promoting ECM synthesis and dermal repair.

### 3.4. Hesperidin Ameliorated Photoaging in Skin Organoids

**Figure 4:**
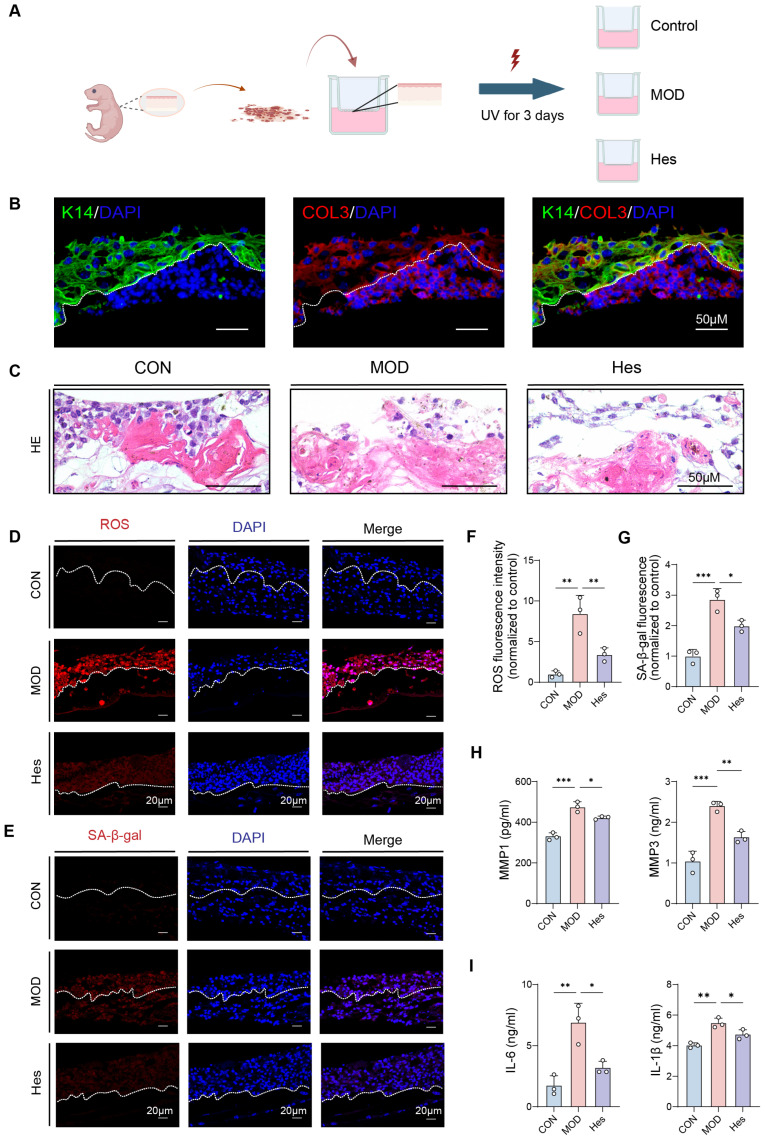
Hesperidin improved photoaging in skin organoids. (**A**) Flowchart of skin organoid establishment and treatment. (**B**) Representative immunofluorescence images of K14 (epidermal marker, green) and COL3 (dermal marker, red) in mouse skin organoids (scale bar = 50 μm). (**C**) Representative HE staining of each group (scale bar = 50 μm). (**D**) ROS staining (ROS, red; DAPI, blue) (scale bar = 20 μm). (**E**) SA-β-gal staining (cardiomyocytes labeled with SA-β-gal antibody, red; DAPI, blue) (scale bar = 20 μm). (**F**) Quantitative analysis of ROS fluorescence (fold with control). (**G**) SA-β-gal fluorescence quantitative analysis (fold with control). (**H**) MMP-1, MMP-3 released by organoids in supernatants; (**I**) IL-1β, IL-6, TNF-α concentration in supernatants. (*n* = 3, * *p* < 0.05, ** *p* < 0.01, *** *p* < 0.001).

To better mimic native skin architecture and cell–cell interactions, we established a 3D mouse skin organoid model (Figure 4A). This 3D model recapitulates the typical dermal-epidermal architecture and biomarker expression pattern of natural skin tissue, providing a physiologically relevant platform for studying photoprotection. Immunofluorescence staining verified successful formation of the dermal-epidermal junction and proper localization of epidermal (K14) and dermal (COL3) markers (Figure 4B), validating the model for photoaging research. Photoaging was induced by UV irradiation (UVA 120 mJ/cm^2^, UVB 80 mJ/cm^2^) for 3 consecutive days. Hematoxylin and eosin (HE) staining showed that the control group exhibited compact and well-organized epidermal and dermal layers. In contrast, the UV-irradiated model group displayed incomplete epidermal differentiation, disordered dermal-epidermal structure, and loose dermis. Hesperidin treatment significantly restored tissue integrity, with morphology similar to the control group (Figure 4C). At the molecular level, UV irradiation markedly increased intracellular oxidative stress and SA-β-gal-positive senescent cells in skin organoids. Notably, hesperidin treatment significantly reduced ROS accumulation (*p* < 0.01) and SA-β-gal activity (*p* < 0.001), indicating suppression of UV-induced oxidative stress and senescence in 3D skin tissue (Figure 4D–G). Extracellular matrix is crucial for maintaining the structural integrity of skin tissue. ELISA results indicated that hesperidin significantly inhibited the UV-induced secretion of the matrix-degrading enzymes MMP-1 (*p* < 0.05) and MMP-3 (*p* < 0.01) (Figure 4H), indicating reduced ECM degradation. Consistent with cellular experiments, hesperidin also suppressed the release of the inflammatory cytokines IL-1β (*p* < 0.05) and IL-6 (*p* < 0.05) (Figure 4I), thereby alleviating SASP. Collectively, these findings demonstrated that hesperidin exerts comprehensive protective effects against UV-induced photoaging in 3D skin organoids. These data further support the anti-photoaging potential of hesperidin and provide reliable preclinical evidence for its future application in humans.

### 3.5. Hesperidin Mitigated Photoaging Progression by Inhibiting Ferroptosis

As the outermost layer of the skin, the epidermis is the first to be affected by UV radiation. Emerging evidence indicates that keratinocytes actively contribute to collagen synthesis, rather than only passively transmitting inflammatory or signaling signals to the dermis [26]. Consequently, UV-induced damage to keratinocytes may directly reduce dermal collagen production and accelerate photoaging. A thorough investigation of the keratinocyte stress response is therefore fundamental to understanding early photoaging events. To explore the underlying mechanism of hesperidin against photoaging, we performed integrated transcriptomic and metabolomic analyses in UV-irradiated HaCaT cells.

**Figure 5:**
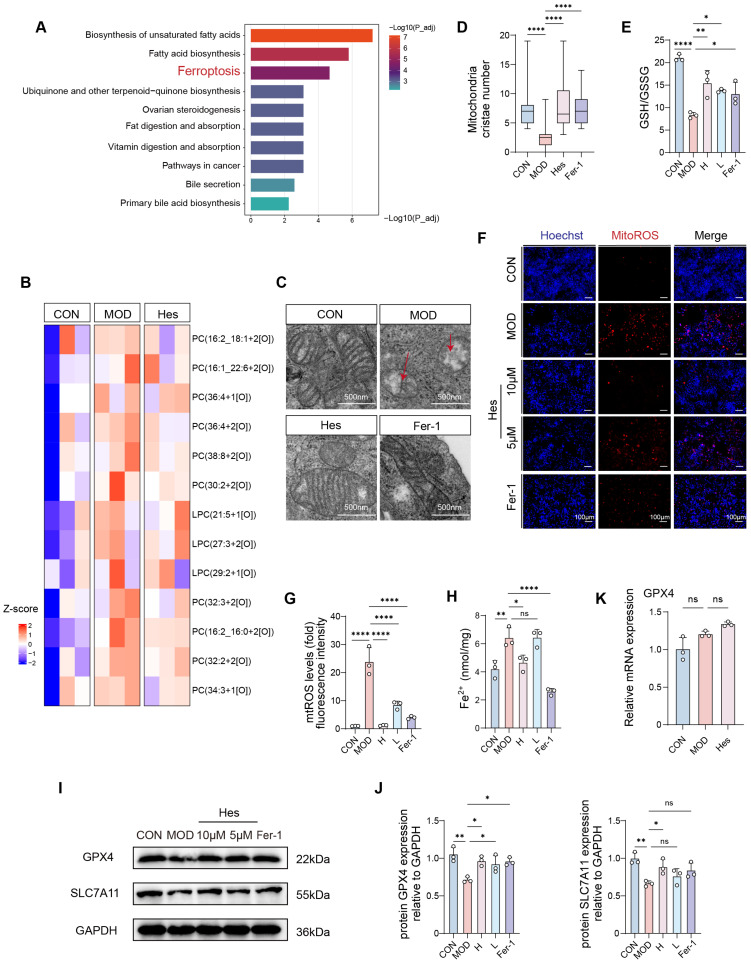
Ferroptosis is a key signaling pathway through which hesperidin ameliorates photoaging in HaCaT cells. (**A**) Metabolite KEGG enrichment analysis. (**B**) Heatmap showing the peroxidation levels of phospholipid in HaCaT cells. phosphatidylcholine (PC), Lysophosphatidylcholine (LPC). Cutoff: log2FC > 0.5, *p* < 0.05 (**C**) Mitochondrial morphology (TEM, scale bar = 500 nm). (**D**) Mitochondrial cristae number of each group. (**E**) GSH/GSSG ratio. (**F**) MitoROS fluorescent staining (mitoROS, red; Hoechst, blue) (scale bar = 100 μm). (**G**) Quantitative analysis of mitoROS fluorescence staining (fold with control). (**H**) Intracellular Fe^2+^ concentration. (**I**,**J**) Western Blot analysis of GPX4 and SLC7A11 protein expression levels. (**K**) Quantitation of mRNA levels of GPX4. (*n* = 3, * *p* < 0.05, ** *p* < 0.01, **** *p* < 0.0001, ns: not statistically significant).

Metabolomic analysis showed that UV irradiation disturbed lipid metabolism, which was notably reversed by hesperidin (Figure S1A). Transcriptomic analysis further indicated that hesperidin regulated lipid metabolism-related pathways (Figure S1F,G). To identify the specific molecular mechanisms involved in these changes, we subsequently performed KEGG enrichment analysis on these differentially metabolized compounds, revealing ferroptosis as the key pathway underlying their altered expression (Figure 5A). Phospholipids serve as the key substrates for lipid peroxidation during ferroptosis, and their oxidative damage directly contributes to cell membrane rupture [27]. Hesperidin decreased the levels of polyunsaturated fatty acid phospholipids (PUFA-PL), including phosphatidylinositol (PI) and phosphatidylethanolamine (PE), compared with the model group (Figure S1B). Levels of polyunsaturated ether phospholipids (PUFA-ePL), which are closely associated with lipid peroxidation and ferroptosis [28], were also reduced (Figure S1C). These findings collectively revealed that hesperidin may reduce the occurrence of UV-induced ferroptosis. Consistent with previous MDA detection results, hesperidin significantly reduced UV-induced PUFA peroxidation (Figure 5B). Mitochondrial morphological abnormality is a core feature of ferroptosis. Transmission electron microscopy revealed that hesperidin mitigated UV-induced mitochondrial morphological damage and cristae disruption (Figure 5C,D). Hesperidin also restored the GSH/GSSG ratio (Figure 5E) and reduced mitochondrial ROS (mitoROS) production (Figure 5F,G) in UV-treated HaCaT cells, similar to the effects of the ferroptosis inhibitor Ferrostatin-1 (Fer-1). The accumulation of intracellular Fe^2+^ is another hallmark of ferroptosis [29]. UV irradiation significantly increased Fe^2+^ levels, which were markedly reduced by hesperidin treatment (Figure 5H). Western blot analysis demonstrated that hesperidin upregulated the expression of key anti-ferroptotic proteins SLC7A11 and GPX4 (Figure 5I,J). Nuclear factor erythroid 2-related factor 2 (Nrf2) is a master regulator of antioxidant pathways, primarily achieving its control over oxidative stress through increased nuclear translocation [30]. Therefore, we also examined intranuclear Nrf2 protein in each group. Results indicated that compared to the control group, Nrf2 protein levels were significantly reduced in the model group. Following hesperidin treatment, Nrf2 protein levels increased relative to the model group (Figure S2A,B), but this increase did not reach statistical significance (*p* > 0.05). To clarify whether reduced GPX4 protein resulted from transcriptional inhibition or enhanced degradation, we detected GPX4 mRNA levels. GPX4 mRNA expression showed no significant change among groups (Figure 5K), indicating that UV-induced GPX4 reduction occurs mainly at the post-translational level. Hesperidin treatment effectively prevented GPX4 protein degradation. These findings collectively demonstrated increased mitochondrial oxidative stress, reduced GPX4 activity, and Fe^2+^ accumulation in the model group constitute essential conditions for driving ferroptosis, with all parameters showing marked improvement following hesperidin treatment. This further confirmed that hesperidin ameliorates photoaging by regulating ferroptosis.

### 3.6. GPX4 Played a Crucial Role in the Regulation of Ferroptosis by Hesperidin

**Figure 6:**
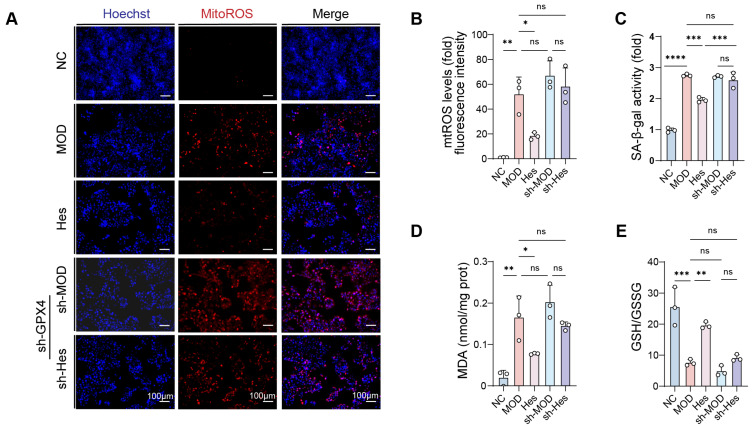
The anti-ferroptotic and anti-photoaging effects of hesperidin are fundamentally dependent on GPX4. (**A**) MitoROS fluorescence staining (mitoROS, red; Hoechst, blue) (scale bar = 100 μm). (**B**) Quantitative analysis of mitoROS fluorescence staining (fold with control). (**C**) SA-β-gal level (fold with control). (**D**) Intracellular MDA level. (**E**) Quantification of GSH/GSSG ratio. (*n* = 3, * *p* < 0.05, ** *p* < 0.01, *** *p* < 0.001, **** *p* < 0.0001, ns: not statistically significant).

We further clarified the molecular mechanism by which hesperidin regulates ferroptosis and exerts anti-photoaging effects. Specifically, we investigated whether these effects are mediated by GPX4 (a key ferroptosis suppressor) or direct regulation of iron ion concentration. To this end, we employed a loss-of-function strategy by stably knocking down GPX4 expression in HaCaT cells using a plasmid-based shRNA approach. In HaCaT cells with GPX4 silenced, the cytoprotective effects of hesperidin against UV-induced ferroptosis and photoaging were significantly attenuated. This abrogation of hesperidin’s protective efficacy confirmed the indispensable role of GPX4 in this pathway. Notably, GPX4 knockdown partially reversed hesperidin’s ability to reduce mitoROS production (Figure 6A,B), decrease SA-β-gal activity (Figure 6C), lower MDA levels (Figure 6D), and increase the GSH/GSSG ratio (Figure 6E). Collectively, these results demonstrate that the anti-ferroptotic and anti-photoaging effects of hesperidin are fundamentally dependent on GPX4, rather than direct regulation of iron ion concentration. This further clarifies the core molecular mechanism underlying hesperidin’s protective effects in UV-damaged HaCaT cells.

### 3.7. HSPA1L Was the Direct Target of Hesperidin in Mitigating Photoaging

**Figure 7:**
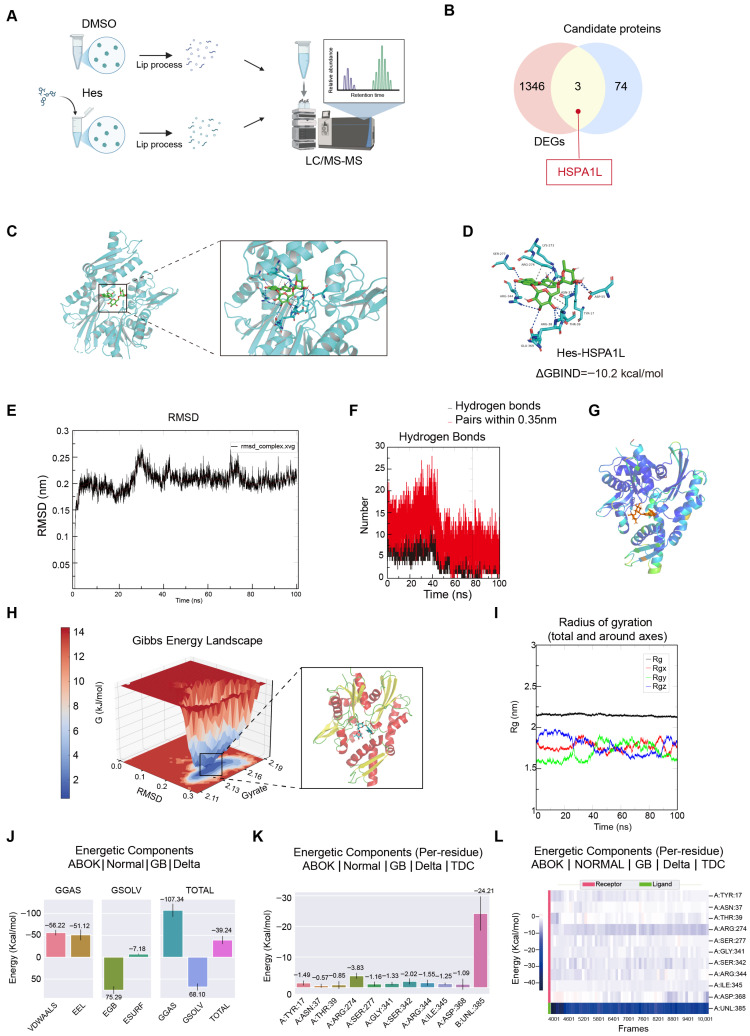
LiP-MS, molecular docking, and molecular dynamics simulations demonstrated HSPA1L as a direct target of hesperidin. (**A**) Lip-MS workflow diagram. (**B**) Venn diagram showing the intersection of differentially expressed genes and proteins. (**C**) Hesperidin-HSPA1L protein binding model and binding pocket visualization. (**D**) Hesperidin-HSPA1L protein binding site. (**E**) RMSD analysis of hesperidin-HSPA1L protein interaction over 100 ns simulation. (**F**) Number of hydrogen bonds in hesperidin-HSPA1L protein binding. (**G**) Visualization of B-factor analysis for hesperidin-HSPA1L complex. (**H**) Gibbs free energy diagram and minimum favored conformation of hesperidin-HSPA1L complex. (**I**) Rg analysis of hesperidin-HSPA1L complex during 100 ns simulation. (**J**–**L**) Binding free energy analysis of the complex.

To elucidate the direct molecular target of hesperidin, we employed an integrated proteomic and computational approach. Limited proteolysis-mass spectrometry (LiP-MS) analysis was first utilized to identify proteins that undergo significant structural changes upon hesperidin binding in a cellular context. This initial screening reveals a set of candidate protein targets (Figure 7A). By integrating this proteomic data with our prior transcriptomic dataset, we performed an intersectional analysis to pinpoint proteins that are differentially expressed at both the protein and mRNA levels. This multi-omics integration narrowed the focus to three high-confidence candidate targets (Figure 7B, Table S3). To evaluate the binding potential of hesperidin to these candidates, we performed molecular docking simulations. Among the tested proteins, the molecular chaperone HSPA1L exhibited the most favorable interaction with hesperidin, demonstrating a strong calculated binding affinity of −10.2 kcal/mol. This robust docking score suggests a highly stable and specific ligand-protein interaction, positioning HSPA1L as the primary candidate for mediating the anti-photoaging effects of hesperidin. Hydrogen bonds were identified between hesperidin and LYS-273, SER-277, ARG-344, GLU-369, ARG-38, THR-39, TYR-17, ASN-37, and ASP-55, while ARG-274 and ASN-37 form hydrophobic interactions (Figure 7C,D). Considering the static nature of molecular docking, we subsequently validated the binding stability of hesperidin with HSPA1L under spatiotemporal conditions using protein-ligand molecular dynamics simulations. Subsequently, we performed root mean square deviation (RMSD) analysis, radial distribution function (RDF), Gibbs free energy plots with visualization of the corresponding minimum-energy structures, radius of gyration (Rg) analysis, B-factor analysis, visualization of MD trajectories at different time points, and MM/GBSA binding free energy calculations on the molecular dynamics simulation results. RMSD analysis revealed that the hesperidin-HSPA1L complex exhibited highly stable trajectories throughout the majority of the simulation period, with minimal fluctuations. Convergence began around 30 ns, indicating the system reached equilibrium (Figure 7E). Hydrogen bond interaction analysis revealed an average of 4.335966 stable hydrogen bonds (bond length < 0.35 nm and hydrogen bond angle < 30°) and an average of 9.961104 unstable hydrogen bonds (bond length criterion satisfied, bond angle criterion unsatisfied), indicating a sufficient total number of hydrogen bonds (Figure 7F). Beta factor analysis (Figure 7G) similarly indicates the stability of the HSPA1L binding pocket. Subsequent analysis of the Gibbs free energy landscape revealed the dominant conformation (Figure 7H) corresponding to the lowest energy state. This dominant conformation is stably located within the docking pocket and is largely consistent with the initial docking pose. Similarly, Rg analysis revealed that the protein in the hesperidin-HSPA1L complex adopts a compact folded conformation (Figure 7I), indicating the stability of the complex. Finally, we employed the MM/GBSA method to calculate the binding energy between hesperidin and HSPA1L. The binding energy of the complex is determined to be −39.24 kcal/mol. We also quantified the binding free energy contributions of individual amino acid residues at the hesperidin–HSPA1L interface (Figure 7J–L). These data indicate that HSPA1L may serve as a direct molecular target of hesperidin in alleviating skin photoaging.

### 3.8. HSPA1L Regulated Ferroptosis by Reducing UV-Induced GPX4 Degradation Through Molecular Chaperone Function

**Figure 8:**
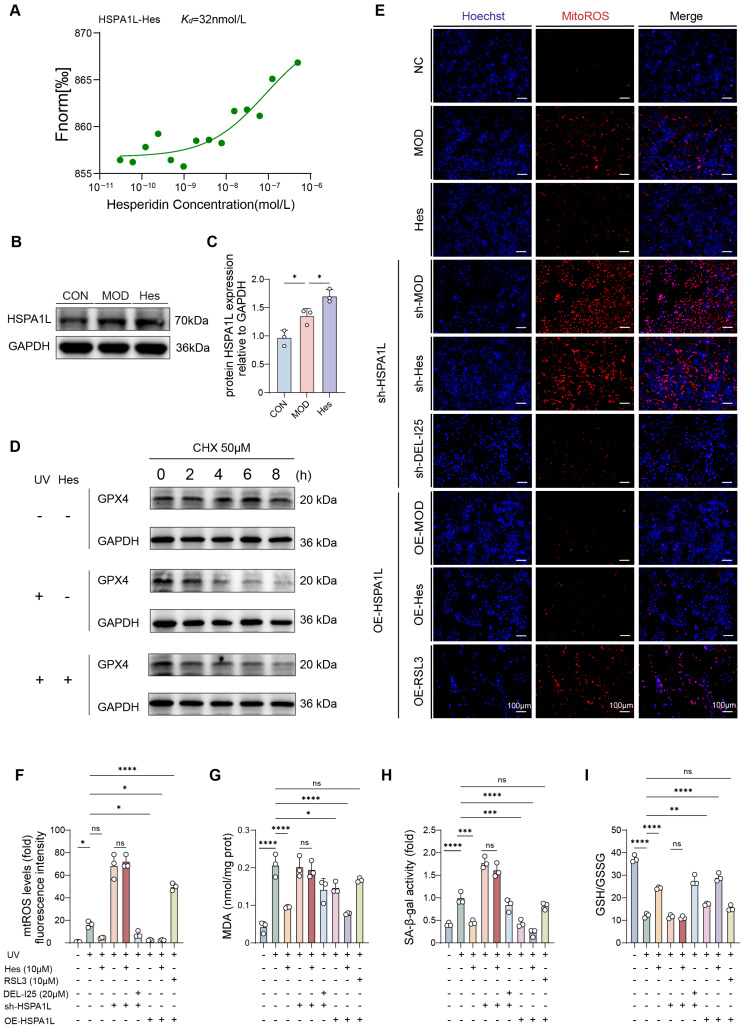
Hesperidin regulated ferroptosis by enhancing protective effect of HSPA1L to GPX4, alleviating photoaging. (**A**) Microscale thermophoresis of HSPA1L protein binding to hesperidin. (**B**) Western Blot of HSPA1L protein expression in HaCaT cells. (**C**) Quantitative analysis of HSPA1L protein. (**D**) Western blot analysis of GPX4 protein degradation following cycloheximide treatment. (**E**) MitoROS fluorescence staining in HaCaT cells (mitoROS, red; Hoechst, blue) (scale bar = 100 μm). (**F**) Quantitative analysis of mitoROS fluorescence staining (fold with control). (**G**) Intracellular MDA level. (**H**) SA-β-gal activity (fold with control). (**I**) Quantification of GSH/GSSG ratio. (*n* = 3, * *p* < 0.05, ** *p* < 0.01, *** *p* < 0.001, **** *p* < 0.0001, ns: not statistically significant).

To biochemically validate the predicted interaction between hesperidin and the molecular chaperone HSPA1L, we performed Microscale Thermophoresis (MST) assays. The results confirmed a direct binding event with a dissociation constant (Kd) of 32 nM, indicating a high-affinity interaction (Figure 8A). We subsequently investigated the functional consequence of this interaction. Under basal conditions, HaCaT keratinocytes exhibited low endogenous levels of HSPA1L. UV irradiation markedly upregulated HSPA1L levels (*p* > 0.05). Notably, hesperidin further significantly elevated HSPA1L protein levels compared to the model group (*p* > 0.05) (Figure 8B,C). These results suggest that hesperidin not only binds to HSPA1L but also potentiates its stress-induced expression.

Previous reports have shown that HSP70 family proteins mitigate UVB-induced epidermal damage through anti-apoptotic, anti-inflammatory, and DNA damage repair mechanisms [31]. Given the known functions of HSP70 proteins, we speculated that HSPA1L upregulation represents a cellular protective response against oxidative stress, and that hesperidin further enhances this response. Based on our previous findings that GPX4 mRNA levels were unchanged, we hypothesize that HSPA1L acts as a molecular chaperone to stabilize GPX4 protein and reduce its degradation. To test this hypothesis, we performed a cycloheximide (CHX, 50 μM) chase assay to monitor GPX4 protein degradation. Results indicated that hesperidin treatment attenuated UV-induced degradation of GPX4 (Figure 8D). To establish the functional link between HSPA1L and GPX4, we constructed HSPA1L-knockdown and HSPA1L-overexpressing HaCaT cells. In cells with HSPA1L knockdown, the inhibitory effects of hesperidin on ferroptosis were abolished, as reflected by increased mitoROS, elevated SA-β-gal activity, higher MDA levels, and a decreased GSH/GSSG ratio. These alterations were reversed upon administration of the GPX4 activator DEL-I25 (20 μΜ). In contrast, HSPA1L overexpression decreased mitoROS, SA-β-gal activity, and MDA levels, while increasing the GSH/GSSG ratio. These protective effects were enhanced by hesperidin but blocked by the GPX4 inhibitor RSL3 (10 μM) (Figure 8E–I). Collectively, these results indicated that HSPA1L regulates ferroptosis by modulating GPX4 protein stability. In conclusion, hesperidin binds to HSPA1L and enhances its expression, which in turn stabilizes GPX4 protein, inhibits ferroptosis, and ultimately mitigates UV-induced skin photoaging.

## 4. Discussion

Skin photoaging is a multifaceted process driven by chronic UV exposure, involving interconnected pathways such as oxidative stress, inflammation, DNA damage, mitochondrial dysfunction, and degradation of the extracellular matrix [32,33]. Although conventional interventions such as antioxidants, retinoids, and laser therapy exert certain protective effects, their clinical applications are often limited by side effects, high cost, or short-term efficacy [34,35,36]. Consequently, sun protection remains the primary strategy for preventing photoaging [37]. These limitations have stimulated growing interest in traditional medicines, especially natural products. Accumulating evidence has demonstrated the anti-photoaging potential of various botanical extracts. Glycyrrhizic acid (GA) and oxymatrine (OMT), for instance, promote collagen and hyaluronic acid synthesis, thereby reducing wrinkle formation and inflammation [38]. Exosomes derived from medicinal fungi like *Phellinus linteus* attenuate UV-induced skin aging by downregulating Mical2 expression [39]. Similarly, extracellular vesicles from *Polygonum* multiflorum alleviate oxidative stress, enhance collagen synthesis, reduce matrix degradation, and mitigate photoaging in human dermal fibroblasts [40]. Notably, Chenpi, a well-known traditional Chinese medicine with a long history of dietary and clinical use, is traditionally used to regulate qi and dispel dampness. Modern pharmacological studies have confirmed its anti-inflammatory and antioxidant activities [41,42]. Hesperidin, one of its major active components, contributes to these effects by regulating inflammatory signaling pathways [43,44].

As the primary bioactive flavonoid of Chenpi, hesperidin demonstrates significant potential in mitigating skin photoaging by enhancing barrier function and protecting against UV radiation-induced damage. Its beneficial effects are largely attributed to its strong antioxidant and anti-inflammatory activities. As a flavonoid, hesperidin possesses multiple phenolic hydroxyl groups that directly scavenge free radicals and reduce oxidative injury [45]. In the present study, hesperidin exerted potent antioxidant effects in HaCaT cells, HSF cells, and skin organoids. These effects included reduced ROS generation, decreased MDA accumulation, and increased SOD activity. Hesperidin also exerted anti-photoaging effects by preserving the extracellular matrix. In our study, hesperidin treatment effectively reduced the expression and activity of MMPs, such as MMP-1 and MMP-3, which are responsible for collagen degradation. In addition, hesperidin reduced senescence-associated markers such as SA-β-gal activity. These findings highlight its ability to maintain skin structural integrity and cellular homeostasis, consistent with previous reports [46]. In addition to direct free radical scavenging, hesperidin regulates key cytoprotective signaling pathways to reinforce cellular defense systems. Previous studies have reported that hesperidin may activate the Nrf2/GPX4 axis and enhance the expression of endogenous antioxidant enzymes [47]. However, in our photoaging model, hesperidin only slightly increased nuclear Nrf2 protein levels, with no statistically significant difference compared with the model group. Consistent with GPX4 mRNA results, hesperidin did not upregulate GPX4 transcription. These results indicate that the Nrf2/GPX4 pathway is not significantly activated by hesperidin in this system. Thus, the protective effects of hesperidin against UV-induced photoaging in HaCaT cells are mediated through other signaling pathways.

Hesperidin exhibited favorable transdermal penetration ability, as confirmed by in vitro transdermal permeation assays. UPLC-MS/MS analysis demonstrated that hesperidin could penetrate the intact skin barrier and reach the underlying skin layers. Results from in vitro experiments showed that hesperidin exerted significant anti-photoaging effects in both the epidermis and dermis. In the epidermis, hesperidin targeted keratinocytes to suppress UV-induced oxidative stress, ferroptosis, and cellular senescence, thereby protecting the outermost skin barrier. In the dermis, hesperidin protected fibroblasts, promoted collagen synthesis, and inhibited extracellular matrix degradation, which is critical for maintaining skin structure and elasticity. Moreover, hesperidin may indirectly protect dermal fibroblasts and promote collagen synthesis by regulating the “epidermal–dermal dialogue”, reducing inflammatory mediators and matrix metalloproteinases derived from keratinocytes. In our study, hesperidin also demonstrated protective effects exerted protective effects in photodamaged skin organoids, confirming its efficacy in a complex 3D skin system. Notably, the present study mainly focused on mechanistic investigations in the epidermis. The crosstalk and regulatory interactions between the epidermis and dermis warrant further exploration.

Ferroptosis is an iron-dependent type of regulated cell death characterized by lipid peroxide accumulation and antioxidant system dysfunction [6,29]. This study revealed that ferroptosis is activated in UV-induced skin photoaging, manifested by decreased GPX4 activity, a reduced GSH/GSSG ratio, elevated MDA levels, accumulation of intracellular Fe^2+^, and distinct mitochondrial damage. Notably, the ferroptosis inhibitor ferrostatin-1 (Fer-1) significantly alleviated these alterations and reduced SA-β-gal activity, confirming ferroptosis as a key driver of photoaging. GPX4 acts as both a structural protein and a major antioxidant enzyme that strongly inhibits lipid peroxidation [11]. It plays roles in lipid and amino acid metabolism [48], influencing cellular aging, tumorigenesis, and cell death. Our results verified that the anti-photoaging effect of hesperidin depends on GPX4. Knockdown of GPX4 abolished the protective effects of hesperidin against photoaging, supporting that the photoprotective action of hesperidin is GPX4-dependent. We further identified HSPA1L as a crucial molecular chaperone in this pathway. Hesperidin enhanced the molecular chaperone function of HSPA1L by increasing its expression, which in turn stabilized GPX4 protein and reduced its degradation. This mechanism parallels previous findings of HSPA5 (GRP78) interacting with GPX4, offering novel insight into the regulation of GPX4 protein stability [49].

This multi-omics analysis provides a systematic overview of the anti-photoaging mechanism of hesperidin. Transcriptomic profiling revealed that hesperidin significantly regulates key genes involved in lipid metabolism. Complementary lipidomic analysis showed that hesperidin drives beneficial remodeling of the lipid profile, particularly by reducing phospholipid species closely associated with ferroptosis. These changes attenuate lipid peroxide accumulation and alleviate UV-induced photoaging damage. A key finding of this study is the identification of HSPA1L as a novel direct molecular target of hesperidin. We further demonstrated that hesperidin upregulates HSPA1L expression, which in turn stabilizes the core anti-ferroptotic enzyme GPX4 and protects it from UV-induced degradation. This effect maintains cellular antioxidant capacity and explains the inhibition of ferroptosis at the molecular level. From a translational perspective, hesperidin presents a promising candidate for developing novel anti-photoaging strategies. To promote its clinical application, future studies should include well-designed clinical trials to rigorously evaluate its efficacy and optimal dosage in humans. In addition, further mechanistic research focusing on the precise binding interface between HSPA1L and GPX4 will be of great value.

Our study demonstrates that hesperidin exerts potent anti-photoaging effects. It inhibits ferroptosis via the HSPA1L-GPX4 axis in human-derived keratinocytes (HaCaTs), fibroblasts (HSF), and a physiologically relevant mouse skin organoid model. The use of human cell lines and the organoid model, which recapitulates key features of native skin architecture, provides reliable preclinical evidence supporting the relevance of this mechanism to human skin. Accordingly, hesperidin is expected to function through a similar pathway in human skin to alleviate UV-induced oxidative stress, cellular senescence, and inflammation. From a translational perspective, hesperidin represents a promising candidate for the development of novel anti-photoaging interventions. However, the translation of in vitro and ex vivo findings to the complex in vivo environment of human skin has inherent limitations. Several aspects require further investigation, including topical formulation design, skin penetration efficiency, metabolism, and long-term safety in humans. Future studies using advanced human skin equivalents or well-designed clinical trials are warranted to validate the efficacy, optimal delivery, and safety of hesperidin as a natural anti-photoaging agent.

In summary, our research identified HSPA1L as a direct target of hesperidin for the first time. We focused on the molecular chaperone function of HSPA1L and its protective effect on GPX4. It offers a novel perspective on regulating GPX4 protein stability, reveals a potential pathway for hesperidin to modulate ferroptosis, and deepens the understanding of the association between ferroptosis, mitochondrial function, and oxidative stress in photoaging. This work establishes a more solid theoretical basis for the intervention of photoaging. It also supports the development of natural anti-photoaging formulations targeting HSPA1L, providing a promising direction for novel skincare products and pharmaceuticals. Despite these important findings, the present study has several limitations. First, the specific structural basis for the interaction between HSPA1L and GPX4 requires further elucidation. We will further verify the direct binding between HSPA1L and GPX4 in future work. Second, the anti-photoaging efficacy of hesperidin in animal models needs to be further confirmed. Third, the precise regulatory role of HSPA1L in ferroptosis should be validated using HSPA1L knockout animals. Finally, we will evaluate the clinical efficacy and safety of hesperidin through clinical trials.

## Figures and Tables

**Table 1:** Chemical Composition of Chenpi in Transdermal Permeation Solution under Negative Ion Mode (Partial).

Component	Area	Formula	Exact Mass	RT
Hesperidin	150203413	C28 H34 O15	610.1985	7.216
Naringin	124133970	C15 H12 O5	271.0663	9.22
Nobiletin	348382	C21 H22 O8	401.1245	8.159
Sinensetin	282767	C20 H20 O7	371.1236	4.738

## Data Availability

The raw RNA-seq data are available in the NCBI Sequence Read Archive (SRA) with the BioProject accession number PRJNA1431559.

## Supplementary Materials

The following supporting information can be downloaded at: https://www.mdpi.com/article/10.3390/antiox15040484/s1, Figure S1. (A) Heatmap showing lipids levels in HaCat cells of each group, Phospholipids (PL), sphingolipids (SP), glycerolipids (GL), fatty acids (FA). (B) Heatmap of individual polyunsaturated fatty acid (PUFA) levels. (C) Heatmap of individual polyunsaturated ether phospholipids (PUFA-ePLs) levels. (D) Heatmap of individual monounsaturated fatty acid (MUFA) levels. (E) Heatmap of individual saturated fatty acid (SFA) levels. (F) Bubble chart of GO enrichment analysis of hesperidin and model group. (G) GSEA enrichment analysis of hesperidin and model group. (Phosphatidylethanolamine (PE), phosphatidylglycerol (PG), phosphatidylinositol (PI), phosphatidylcholine (PC), phosphatidylserine (PS), Phosphatidic Acid (PA), Lysophosphatidylethanolamine (LPE/LysoPE), Lysophosphatidylglycerol (LPG/LysoPG), Lysophosphatidylserine (LPS), Lysophosphatidylinositol (LPI), Cutoff: log2FC > 0.5 and *p* < 0.05, *n* = 3). Figure S2. (A) Western Blot of Nrf2 protein in HaCaT cells. (B) Quantitative analysis of Nrf2 protein. (*n* = 3, * *p* < 0.05, ns: not statistically significant). Table S1. Primer list. Table S2. Plasmid sequence. Table S3. Candidate targets.

## Abbreviations

The following abbreviations are used in this manuscript:

UV	Ultraviolet
HSPA1L	Heat Shock protein 70 A Like.
RT-qPCR	Reverse Transcription Quantitative polymerase chain reaction
HE	Hematoxylin and eosin (staining)
ELISA	Enzyme linked immunosorbent assay
LiP-MS	Limited proteolysis coupled with mass spectrometry
MST	Microscale thermophoresis
GPX4	Glutathione peroxidase 4
SLC7A11	Solute Carrier Family 7 Member 11
AGEs	Advanced glycation end products
MMP-1	Matrix Metalloproteinase-1
MMP-3	Matrix Metalloproteinase-3
MMP-9	Matrix Metalloproteinase-9
ROS	Reactive Oxygen Species
MDA	Malondialdehyde
SOD	Superoxide dismutase
SA-β-gal	Senescence-associated beta-galactosidase
HaCaT	Human adult low Calcium high Temperature keratinocyte (cell)
HSF	Human Skin Fibroblast (cell)
ATG5	Autophagy Related Protein 5
HMGB1	High Mobility Group Box 1
IFN-γ	Interferon-gamma
HSP70	Heat shock protein 70
UPLC-MS/MS	Ultra Performance Liquid Chromatography Tandem Mass Spectrometry
Nrf2	Nuclear factor erythroid 2-related factor 2
ECM	extracellular matrix
VitE	Vitamin E
IL-1β	Interleukin-1β
IL-6	Interleukin-6
TNF-α	Tumor Necrosis Factor-alpha
DAPI	4′ 6-diamidino-2-phenylindole
mitoROS	Mitochondrial Reactive Oxygen Species
TEM	Transmission electron microscopic
PFA	Paraformaldehyde
CHX	Cycloheximide
Fer-1	Ferrostatin-1
GSH	Glutathione
GSSG	Glutathione disulfide
GA	Glycyrrhizic acid
OMT	oxymatrine
PUFA	polyunsaturated fatty acid
PE	phosphatidylethanolamine
PI	phosphatidylinositol
PL	phospholipid
CCK-8	Cell counting kit-8
SASP	Senescence-associated secretory phenotype
