# Supplementary material for: Hesperidin from Chenpi Ameliorates Skin Photoaging by Targeting HSPA1L to Stabilize GPX4 and Suppress Ferroptosis
Source: Antioxidants (Basel). 2026 Apr 14;15(4):484. doi: 10.3390/antiox15040484 (PMC13114056; doi:10.3390/antiox15040484)
Supplement: Supplementary file 1 [file antioxidants-15-00484-s001.zip › antioxidants-4174266-supplementary.pdf]

## Supplementary Material

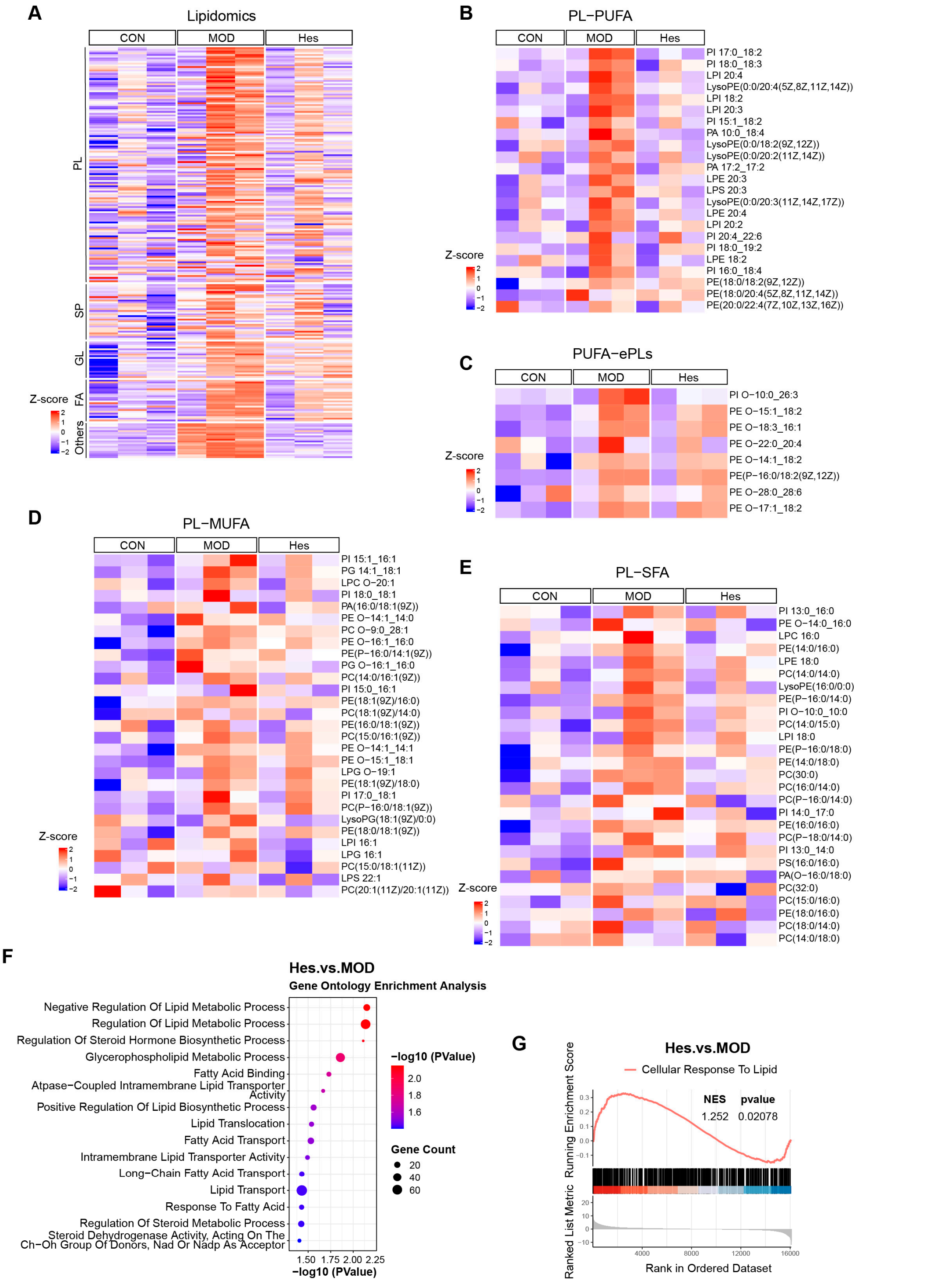


Fig. S1. (A) Heatmap showing lipids levels in HaCat cells of each group, Phospholipids (PL), sphingolipids (SP), glycerolipids (GL), fatty acids (FA). (B) Heatmap of individual polyunsaturated fatty acid (PUFA) levels. (C) Heatmap of individual polyunsaturated ether phospholipids (PUFA-ePLs) levels. (D) Heatmap of individual monounsaturated fatty acid (MUFA) levels. (E) Heatmap of indivi

dual saturated fatty acid (SFA) levels. (F) Bubble chart of GO enrichment analysis of hesperidin and model group. (G) GSEA enrichment analysis of hesperidin and model group. (Phosphatidylethanolamine (PE), phosphatidylglycerol (PG), phosphatidylinositol (PI), phosphatidylcholine (PC), phosphatidylserine (PS), Phosphatidic Acid (PA), Lysophosphatidylethanolamine (LPE/LysoPE), Lysophosphatidylglycerol (LPG/ LysoPG), Lysophosphatidylserine (LPS), Lysophosphatidylinositol (LPI), Cutoff: log2FC>0.5 and $P<0.05$, n=3)

Fig. S2.

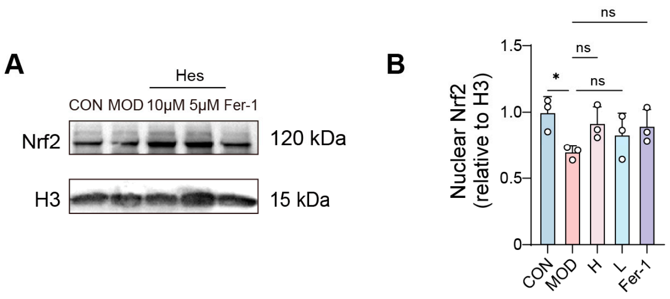


**Figure S2.** (A) Western Blot of Nrf2 protein in HaCaT cells. (B) Quantitative analysis of Nrf2 protein. (n = 3, $*p < 0.05$, ns: not statistically significant)

| Gene | Species | Primers |
|---|---|---|
| IL-1β | Mouse | Forward: GCCCCAAAGAGATGAAGTGC<br>Reverse: TTTCAAGGACGATGGGCTCT |
| IL-6 | Mouse | Forward: CAAAGCCAGAGTCCTTCAGAG<br>Reverse: AGCATTGGAAATTGGGGTAG |
| TNF-α | Mouse | Forward: TCTTCTCATTCCTGCTTGTGG<br>Reverse: ATGAGAGGGAGGCCATTTG |
| COL1A1 | Mouse | Forward: TCAGAGGCGAAGGCAACAGTC<br>Reverse: GCAGGCGGGAGGTCTTGG |
| COL3 | Mouse | Forward: GTCCACAAGGATTACAAGGCATACC<br>Reverse: CCAGGAGCACCGACTTCACC |
| MMP1 | Mouse | Forward: CAGTTGACAGGCTCCGAGAAATG<br>Reverse: CACATCAGGCACTCCACATCTTG |
| MMP3 | Mouse | Forward: GACGATGATGAACGATGGACAGAG<br>Reverse: GCCTTGGCTGAGTGGTAGAGTC |
| TIMP1 | Mouse | Forward: GCATCTCTGGCATCTGGCATCC<br>Reverse: GTCCGAGTTGCAGAAGGCTGTC |
| GAPDH | Mouse | Forward: GGTTGTCTCCTGCGACTTCA<br>Reverse: TGGTCCAGGGTTTCTTACTCC |

| | | |
|---|---|---|
| MMP1 | Human | Forward: TTACACGCCAGATTTGCCAAGAG<br>Reverse: TCAGAGGTGTGACATTACTCCAGAG |
| MMP3 | Human | Forward: GGTCTCTTTCACTCAGCCAACAC<br>Reverse: CAGGCGGAACCGAGTCAGG |
| TIMP1 | Human | Forward: CCTGTTGTTGCTGTGGCTGATAG<br>Reverse: CTGATGACGAGGTCGGAATTGC |
| GAPDH | Human | Forward: CAGGAGGCATTGCTGATGAT<br>Reverse: GAAGGCTGGGGCTCATTT |

Table S1 Primer list

| shRNA Name | Sense（5'-3'） | Antisense（5'-3'） |
|---|---|---|
| *GPX4-sh1* | CACCGCCGCCTTTGCCGCCTACTGACGAATCAGTAGGCGGCAAAGGCGGC | CGGCGGAAACGGCGGATGACTGCTTAGTCATCCGCCGTTTCCGCCGAAAA |
| *GPX4-sh2* | CACCGCCTTTGCCGCCTACTGAAGCCGAAGCTTCAGTAGGCGGCAAAGGC | CGGAAACGGCGGATGACTTCGGCTTCGAAGTCATCCGCCGTTTCCGAAAA |
| *GPX4-sh3* | CACCGGACACCGTCTCTCCACAGTTCGAAAACTGTGGAGAGACGGTGTCC | CCTGTGGCAGAGAGGTGTCAAGCTTTTGACACCTCTCTGCCACAGGAAAA |
| *GPX4-sh4* | CACCGACACCGTCTCTCCACAGTTCCGAAGAACTGTGGAGAGACGGTGTC | CTGTGGCAGAGAGGTGTCAAGGCTTCTTGACACCTCTCTGCCACAGAAAA |
| *HSPA1L-sh1* | CACCGCCAAGAACCAGGTAGCAATGCGAACATTGCTACCTGGTTCTTGGC | CGGTTCTTGGTCCATCGTTACGCTTGTAACGATGGACCAAGAACCGAAAA |
| *HSPA1L-sh2* | CACCGCTTTCTACCCTGAGGAAATCCGAAGATTTCCTCAGGGTAGAAAGC | CGAAAGATGGGACTCCTTTAGGCTTCTAAAGGAGTCCCATCTTTCGAAAA |
| *HSPA1L-sh3* | CACCGCTGGACTTAATGTGCTAAGACGAATCTTAGCACATTAAGTCCAGC | CGACCTGAATTACACGATTCTGCTTAGAATCGTGTAATTCAGGTCGAAAA |
| *HSPA1L-sh4* | CACCGGACTTAATGTGCTAAGAATCCGAAGATTCTTAGCACATTAAGTCC | CCTGAATTACACGATTCTTAGGCTTCTAAGAATCGTGTAATTCAGGAAAA |

Table S2 Plasmid sequence

| Overlap | Docking energy (kcal/mol) | Molecular weight (kDa) |
|---|---|---|
| NDUFA11 | -7.2 | 19 kDa |
| HSPA1L | -10.2 | 70 kDa |
| ABAT | -8.6 | 42 kDa |

Table S3 Candidate targets
